# Supplementary material for: Feasibility Testing of the Health4LIFE Weight Loss Intervention for Primary School Educators Living with Overweight/Obesity Employed at Public Schools in Low-Income Settings in Cape Town and South Africa: A Mixed Methods Study
Source: Nutrients. 2024 Sep 11;16(18):3062. doi: 10.3390/nu16183062 (PMC11435216; doi:10.3390/nu16183062)
Supplement: Supplementary file 1 [file nutrients-16-03062-s001.zip › Supplementary Table S1.pdf]

Table S1: Outline of the Health4LIFE intervention weight loss manual and linked text messages

Chapter title in manual	Content outline of manual	Self-directed activities in the manual	Text messages directed at manual content
**Chapter 1:**<br>Weight management	Weight balance<br><br>Energy balance<br><br>Health benefits associated with weight loss for those who are overweight or obese	**Chapter 1:**<br>Energy balance assessment<br><br>**Addendum 3:**<br>Assessment of BMI, WC, risk factors for disease and conditions associated with obesity;<br>Weight loss goal setting;<br>Weight monitoring chart	For the first week text messages focused on general weight management information
**Chapter 2:**<br>General healthy eating advice	South African FBDG and food guide<br><br>DASH eating plan	**Chapter 2:**<br>Brief fruit and vegetable intake assessment; fat intake assessment; sugar intake assessment	N/A
**Chapter 3:**<br>Fruit and vegetables	FBDG (stating the guideline)<br><br>General information (recommendations to increase fruit and vegetables intake, serving sizes, explaining eating a variety)<br><br>Health benefits of eating fruit and vegetables<br><br>Action plan (outlining the target behaviours: purchase a variety of fruit and vegetables, bring fruit and vegetables to school every day, eat a variety of vegetables at mealtimes every day)<br><br>Problem solving (tips to buy fruit and vegetables on a restricted budget, lunchbox ideas, tips to meet the daily recommendation, cooking methods for vegetables)	**Addendum 3:**<br>Assessment of current fruit and vegetable intake and goal setting;<br>Weekly record sheet of variety of fruit and vegetables purchased and served at mealtimes	There were three target behaviours related to fruit and vegetable intake which the text messages targeted over a three-week period:<br>• Purchase a variety of fruit and vegetables<br>• Bring fruit and vegetables to school every day<br>• Eat a variety of vegetables at mealtimes
**Chapter 4:**<br>Fat	FBDG (stating the guideline)<br><br>General information (what are fats, the types of fat, fat intake and weight loss)<br><br>Health benefits of controlling fat intake	**Addendum 3:**<br>Assessment of current fat intake and goal setting;<br>Weekly record sheet of fat intake during meal preparation and selecting snacks	There were three target behaviours related to fat intake which the text messages targeted over a five-week period:<br>• Select food low in fat when purchasing foods<br>• Add less fat when preparing food<br>• Reduce or avoid the intake of high fat snacks

	Action plan (outlining the target behaviours: select foods low in fat when purchasing foods, add less fat when preparing foods, reduce or avoid high fat snacks or processed foods)<br><br>Problem solving (shopping list for lower fat options, lower fat recipe substitutes, low fat options when eating out, tips to reduce fat when cooking, low fat snack ideas)		
**Chapter 5:**<br>Sugar	FBDG (stating the guideline)<br><br>General information (what is sugar)<br><br>Health benefits of decreasing sugar intake<br><br>Action plan (outlining the target behaviours: limit the purchase of sugar containing foods and drinks, reduce or avoid the intake of sugar-containing cold drinks, add less or no sugar to hot beverages, use less or no sugar when preparing foods, avoid the intake of sugary foods/snacks during times of stress)<br><br>Problem solving (sugar content in breakfast cereals, hidden sugar in foods, reducing the sugar in cold drinks, rethink your drink, tips to add less sugar to hot beverages, side-effects related of decreasing sugar intake)	**Addendum 3:**<br>Assessment of current sugar intake and goal setting;<br>Weekly record of sugar intake during meal preparation, in beverages and at times of stress	There were five target behaviours related to sugar intake which the text messages targeted over a five-week period:<br>• Limit the purchase of sugar-containing food/drinks<br>• Reduce or avoid the intake of sugar-containing cold drinks<br>• Use less or no sugar when preparing foods<br>• Add less or no sugar to hot beverages<br>• Avoid the intake of sugary foods/drinks during times of stress
**Chapter 6:**<br>Physical activity	FBDG (stating the guideline)<br><br>General information (physical activity recommendations, types of physical activity, levels of intensity)<br><br>Health benefits of physical activity<br><br>Action plan (outlining the target behaviours: increase physical activity at work, increase physical activity in free time)<br><br>Problem solving (getting started and staying active, practical tips to build activity into daily routine, typical excuses not to exercise and possible solutions, drinking during exercise)	**Addendum 3:**<br>Assessment of current level of physical activity and goal setting;<br>Weekly record of physical activities at school and during free time	Each week one text message targeted the physical activity targeted behaviours:<br>• Increase physical activity at work<br>• Increase physical activity in free time
**Chapter 7**:<br>Stress management	General information (what is stress, health implications, typical stressors for educators)	**Addendum 3:**<br>Stress indicator checklist;<br>Goal setting;	Text messages targeting stress management was incorporated into the week where the target behaviour "Avoid the intake of sugary foods/drinks during times of stress" was addressed

	Action plan (9 actions to improve stress management skills including relaxation techniques and exercise)	Weekly record of management of stress	
**Addendum 1:** Eating plans	Examples of various caloric eating plans: 1000kcal, 1200kcal, 1400kcal, 1600kcal, 1800kcal	N/A	
**Addendum 2:** Useful tips and information	Time-saving cooking tips and lunch tips;<br><br>Cooking tips to reduce added sugar;<br><br>Snacks containing sugar;<br><br>Food label reading	N/A	At least once a week, educators were referred to tips and information in the manual
**Addendum 3:** Self-assessment, goal setting and self-monitoring activities	Assessment of current:<br>Weight status and health risk<br>Fruit and vegetable intake<br>Fat intake<br>Sugar intake<br>Physical activity<br>Stress<br><br>Goal setting for:<br>Weight<br>Fruit and vegetable intake<br>Fat intake<br>Sugar intake<br>Physical activity<br>Stress management<br><br>Weekly monitoring of:<br>Weight<br>Fruit and vegetable intake<br>Fat intake<br>Sugar intake<br>Physical activity<br>Stress management	N/A	At the end of each week, the text message referred the educator to the activities related to the target behaviour which was the focus of the week

NA: Not applicable, DASH: Dietary Approaches To Stop Hypertension [Appel et al., 1997], FBDG: Food Based Dietary Guidelines [Vorster et al., 2013]; kcal: kilocalories; WC: Waist circumference
