# Supplementary material for: Feasibility Testing of the Health4LIFE Weight Loss Intervention for Primary School Educators Living with Overweight/Obesity Employed at Public Schools in Low-Income Settings in Cape Town and South Africa: A Mixed Methods Study
Source: Nutrients. 2024 Sep 11;16(18):3062. doi: 10.3390/nu16183062 (PMC11435216; doi:10.3390/nu16183062)
Supplement: Supplementary file 1 [file nutrients-16-03062-s001.zip › Supplementary Table S2.pdf]

Table S2: Interview guide for the in-depth semi-structured interviews for educators

Topic	Discussion
Introduction	Interviewer's name
Topic of interview	Today I would like to discuss the weight loss intervention you participated in.
Aim of interview and responses	There are no right or wrong answers to any of the questions. I am wanting to gain insights and information about your experience with this weight loss intervention in order to modify and improve future interventions of this nature.
Explaining note-taking and tape recording	I (interviewer's name) will be taking notes during our discussion to help with the understanding of the information provided by you. I also would like to use a tape recorder to ensure that your answers are accurately recorded. Are you happy with this?
Check understanding	Do you understand?
Clarification needed	Do you have any questions?
Elicitation questions	
1. Why were you interested in this intervention?	
2. What would it mean to you if you lost weight?	
3. What did the people around you (eg. Family, friends, colleagues) think about you trying to lose weight?	
4. What made it easy for you to follow the intervention?	
5. What made it difficult for you to follow the intervention?	
6. What did you like about the content of the manual?<br>-Content layout, writing size (font) and amount of information?	
7. What did you not like about the content of the manual?<br>Prompts -content layout, writing size (font) and amount of information?	
8. What information in the manual did you find useful?<br>Prompt -Why?	
9. What information in the manual was not useful?<br>Prompt-Why?	
10. Was there anything in the content of the manual which you did not understand?<br>Prompt-What made it difficult to understand?	
11. What did you think about the activities (examples: self-assessment, goal setting and self-monitoring) that were included in the manual?<br>Prompts -Did you complete any of the activities? If no, why?<br>-If yes, which ones did you complete, why did you complete them and how helpful were they	
12. What did you think about the eating plans provided in the manual?<br>Prompt-Useful, appropriate?	
13. What did you think about the tips and information provided in the manual?<br>Prompt-Useful, appropriate, give examples?	
14. What did you think about the mobile phone messages you received?<br>Prompts –The content?<br>The timing?<br>-To what extent do you think the messages contributed to participation in the intervention<br>-How did it encourage you OR why did it not encourage you?	
15. Explain how you experienced the intervention as a whole?	

-Are there any tips you can give us to improve the intervention?	
Closing points	So in summary you are saying ……….<br>Is there anything more you would like to add?<br>Thank you for your time!
