# Supplementary material for: Feasibility Testing of the Health4LIFE Weight Loss Intervention for Primary School Educators Living with Overweight/Obesity Employed at Public Schools in Low-Income Settings in Cape Town and South Africa: A Mixed Methods Study
Source: Nutrients. 2024 Sep 11;16(18):3062. doi: 10.3390/nu16183062 (PMC11435216; doi:10.3390/nu16183062)
Supplement: Supplementary file 1 [file nutrients-16-03062-s001.zip › Supplementary Table S3.pdf]

Table S3: Interview guide for the in-depth semi-structured interview for principals

Topic	Discussion
Introduction	Interviewer's name
Topic of interview	Today I would like to discuss the weight loss intervention your school participated in.
Aim of interview and responses	There are no right or wrong answers to any of the questions. I am wanting to gain insights and information about your experience with how this weight loss intervention was implemented in order to modify and improve future interventions of this nature.
Explaining note-taking and tape recording	I (interviewer's name) will be taking notes during our discussion to help with the understanding of the information provided by you. I also would like to use a tape recorder to ensure that your answers are accurately recorded. Are you happy with this?
Check understanding	Do you understand?
Clarification needed	Do you have any questions?
Elicitation questions	
1. Why were you interested in allowing your school to participate in this intervention?	
2. What did you think about having the wellness day at your school?	
3. What did the educators think about having the wellness day at your school?	
4. What made it easy for you to say yes to the wellness day?	
5. What made it difficult for you to say to the wellness day?	
6. What did you think about having the intervention at your school?	
7. What did the educators think about having the intervention at your school?	
8. What made it easy for you to say yes to the intervention?	
9. What made it difficult for you to say yes to the intervention?<br>Prompt – did you allow the 16-week follow-up? If no, why?	
10. Explain how you experienced the intervention as a whole?<br>-Are there any tips you can give us to improve the implementation process of the intervention?	
Closing points	So in summary you are saying ……….<br>Is there anything more you would like to add?<br>Thank you for your time!
