# Supplementary material for: Feasibility Testing of the Health4LIFE Weight Loss Intervention for Primary School Educators Living with Overweight/Obesity Employed at Public Schools in Low-Income Settings in Cape Town and South Africa: A Mixed Methods Study
Source: Nutrients. 2024 Sep 11;16(18):3062. doi: 10.3390/nu16183062 (PMC11435216; doi:10.3390/nu16183062)
Supplement: Supplementary file 1 [file nutrients-16-03062-s001.zip › Supplementary Table S4a anf S4b.pdf]

Table S4(a): Baseline belief patterns retrieved from the belief data for the intervention group

No	Belief statement	Pattern 1	Pattern 2	Pattern 3
24	I can increase my levels of physical activity (be physically more active).	**0.85***	0.09	0.01
8	Decreasing the amount of fat I eat will help me lose/ control my weight.	**0.84***	0.09	-0.20
23	Having an exercise 'buddy' will help me to be physically more active.	**0.82***	0.07	-0.14
21	Knowing more about different types of physical activity I can do will help me to be more active.	**0.74***	0.30	0.06
6	Fruit and vegetables are easy to find in stores nearby.	**0.74***	-0.03	0.26
19	Being physically more active will make me feel better about my appearance.	**0.69***	0.39	0.11
17	I can reduce the amount of sugary foods/snack/drinks I eat and drink.	**0.66***	-0.12	0.01
14	I turn to sugary foods/snacks/drinks when I am stressed.	**0.60***	0.15	0.37
22	I could be physically more active even if I were tired.	**0.60***	0.48*	0.06
20	Finding time to be physically more active is possible.	**0.54***	0.48*	0.26
7	Eating less fat will help reduce the risk of diseases e.g. heart disease.	**0.54***	-0.15	-0.10
2	Eating fruits and vegetables every day will help me lose weight/ control my weight.	**0.52***	0.25	-0.25
13	Low fat/ fat-free foods taste good/ are tasty.	0.12	**0.74***	-0.18
11	It is easy to exclude high-fat foods from my daily diet.	0.27	**0.62***	0.19
12	I do not have enough time to prepare healthy meals regularly.	-0.18	**0.56***	-0.07
15	I have poor awareness of the sugar content in the foods/snacks/drinks I eat and drink.	-0.02	0.07	0.81*
10	Healthy takeaways and/or street foods are easy to find in my surroundings.	0.19	-0.04	**0.75***
16	Reducing the amount of sugary foods/snacks/drinks I eat and drink will make me feel unwell (moody or have a headache or tired).	-0.03	0.49*	**0.56***
1	Preparation of vegetables does not take a long time.	0.27	0.17	**-0.49***

Beliefs which did not load on any factor: 3; 4; 5; 9; 18

Fat intake beliefs | Fruit and vegetable intake beliefs | Sugar intake beliefs

Physical activity beliefs | Health effects of healthy lifestyle beliefs | Generic beliefs

% Variance explained by each pattern: Pattern 1 31.1 % | Pattern 2 12.5% | Pattern 3 11.9%

Kaiser's Measure of Sampling Adequacy: 0.62

Table S4(b): Change in belief patterns from baseline to 16-week follow-up retrieved from the belief data for the Intervention completers

No	Belief statement	Pattern 1	Pattern 2	Pattern 3
3	Fruits and vegetables are affordable.	**0.80***	-0.01	-0.24
24	I can increase my levels of physical activity (be physically more active)	**0.79***	0.13	0.16
21	Knowing more about different types of physical activity I can do will help me to be more active.	**0.76***	0.17	0.09
4	I can eat the recommended amount of fruits and vegetables every day.	**0.71***	0.15	-0.09
17	I can reduce the amount of sugary foods/snack/drinks I eat and drink	**0.63***	0.32	0.11
20	Finding time to be physically more active is possible.	**0.62***	0.08	-0.05
7	Eating less fat will help reduce the risk of diseases e.g. heart disease.	**0.57***	0.56	-0.24
23	Having an exercise 'buddy' will help me to be physically more active.	**0.56***	0.46*	0.22
5	I would eat vegetables even if at times, they look unappealing.	**0.56***	0.09	-0.12
6	Fruit and vegetables are easy to find in stores nearby	**0.54***	0.33	-0.18
11	It is easy to exclude high-fat foods from my daily diet.	**0.45***	0.31	-0.29
2	Eating fruits and vegetables every day will help me lose weight/ control my weight.	0.16	**0.86***	0.01
8	Decreasing the amount of fat I eat will help me lose/ control my weight.	0.47	**0.66***	-0.15
12	I do not have enough time to prepare healthy meals regularly.	-0.02	**0.63***	0.08
19	Being physically more active will make me feel better about my appearance.	0.53	**0.62***	0.05
18	There are no accessible, safe, affordable opportunities for me to be physically active.	0.05	0.15	**0.71***
16	Reducing the amount of sugary foods/snacks/drinks I eat and drink will make me feel unwell (moody or have a headache or tired).	-0.07	-0.22	**0.63***
9	Low-fat/healthy fat options are expensive.	-0.31	0.36	**0.62***
22	I could be physically more active even if I were tired.	0.49*	-0.04	**0.54***

Beliefs which did not load on any factor: 1; 10; 13; 14; 15

Fat intake beliefs | Fruit and vegetable intake beliefs | Sugar intake beliefs

Physical activity beliefs | Health effects of healthy lifestyle beliefs | Generic beliefs

% Variance explained by each pattern: Pattern 1 28.9% | Pattern 2 16.3% | Pattern 3 10.5%

Kaiser's Measure of Sampling Adequacy: 0.70
