# Supplementary material for: Feasibility Testing of the Health4LIFE Weight Loss Intervention for Primary School Educators Living with Overweight/Obesity Employed at Public Schools in Low-Income Settings in Cape Town and South Africa: A Mixed Methods Study
Source: Nutrients. 2024 Sep 11;16(18):3062. doi: 10.3390/nu16183062 (PMC11435216; doi:10.3390/nu16183062)
Supplement: Supplementary file 1 [file nutrients-16-03062-s001.zip › Supplementary Table S5a and S5b.pdf]

Table S5(a): Baseline belief patterns retrieved from the belief data for the control group

No	Belief statement	Pattern 1	Pattern 2	Pattern 3
8	Decreasing the amount of fat I eat will help me lose/ control my weight.	**0.78***	-0.29	0.24
2	Eating fruits and vegetables every day will help me lose weight/ control my weight.	**0.75***	0.20	-0.15
4	I can eat the recommended amount of fruits and vegetables every day.	**0.61***	0.24	0.17
7	Eating less fat will help reduce the risk of diseases e.g. heart disease.	**0.57***	-0.13	0.40
3	Fruits and vegetables are affordable.	**0.55***	0.19	-0.17
17	I can reduce the amount of sugary foods/snack/drinks I eat and drink.	**0.48***	0.39	-0.21
11	It is easy to exclude high-fat foods from my daily diet.	0.27	**0.79***	0.05
13	Low fat/ fat-free foods taste good/ are tasty.	0.36	**0.63***	0.18
6	Fruit and vegetables are easy to find in stores nearby.	0.03	**0.61***	0.09
20	Finding time to be physically more active is possible.	0.11	**0.53***	-0.18
12	I do not have enough time to prepare healthy meals regularly.	0.22	**-0.69***	0.04
23	Having an exercise 'buddy' will help me to be physically more active.	-0.07	0.01	**0.79***
5	I would eat vegetables even if at times, they look unappealing.	0.33	0.04	**0.58***
15	I have poor awareness of the sugar content in the foods/snacks/drinks I eat and drink.	0.09	-0.01	**-0.73***

Beliefs which did not load on any factor: 1; 9; 10; 14; 16; 18; 19; 21; 22; 24

Fat intake beliefs   Fruit and vegetable intake beliefs   Sugar intake beliefs

Physical activity beliefs   Health effects of healthy lifestyle beliefs   Generic beliefs

% Variance explained by each pattern: Pattern 1 20.0%   Pattern 2 18.2%   Pattern 3 13.6%

Kaiser's Measure of Sampling Adequacy: 0.64

Table S5(b): Change in belief patterns from baseline to 16-week follow-up retrieved from the belief data for the control completers

No	Belief statement	Pattern 1	Pattern 2	Pattern 3
7	Eating less fat will help reduce the risk of diseases e.g. heart disease.	**0.86***	0.16	0.23
8	Decreasing the amount of fat I eat will help me lose/ control my weight.	**0.78***	0.14	0.22
9	Low-fat/healthy fat options are expensive.	**0.67***	-0.27	-0.02
2	Eating fruits and vegetables every day will help me lose weight/ control my weight.	**0.52***	0.25	0.35
21	Knowing more about different types of physical activity I can do will help me to be more active.	0.06	**0.87***	0.16
20	Finding time to be physically more active is possible.	-0.08	**0.80**	0.07
19	Being physically more active will make me feel better about my appearance.	0.50*	**0.66***	-0.18
24	I can increase my levels of physical activity (be physically more active)	0.20	**0.51***	0.20
23	Having an exercise 'buddy' will help me to be physically more active.	0.41	**0.48***	-0.09
5	I would eat vegetables even if at times, they look unappealing.	0.29	**-0.49***	0.20
3	Fruits and vegetables are affordable.	-0.28	-0.14	**0.72***
4	I can eat the recommended amount of fruits and vegetables every day.	0.10	0.28	**0.68***
1	Preparation of vegetables does not take a long time.	0.22	-0.01	**0.62***
6	Fruit and vegetables are easy to find in stores nearby	0.38	-0.27	**0.61***
11	It is easy to exclude high-fat foods from my daily diet.	0.33	0.25	**0.49***

Beliefs which did not load on any factor: 10; 12; 13; 14; 15; 16; 17; 18; 22

Fat intake beliefs   Fruit and vegetable intake beliefs

Physical activity beliefs   Health effects of healthy lifestyle beliefs

% Variance explained by each pattern: Pattern 1 20.0%   Pattern 2 19.8%   Pattern 3 15.7%

Kaiser's Measure of Sampling Adequacy: 0.63
